# The Impact of Child Behaviour Problems on Maternal Employment: A Longitudinal Cohort Study

**DOI:** 10.1007/s10834-013-9378-8

**Published:** 2013-10-31

**Authors:** Ragnhild Bang Nes, Lars Johan Hauge, Tom Kornstad, Petter Kristensen, Markus A. Landolt, Leif T. Eskedal, Lorentz M. Irgens, Margarete E. Vollrath

**Affiliations:** 1 Division of Mental Health, Norwegian Institute of Public Health, PO Box 4404, Nydalen, 0403 Oslo, Norway; 2 Institute of Psychology, University of Oslo, Oslo, Norway; 3 Statistics Norway, Oslo, Norway; 4 University Children’s Hospital, Zurich, Switzerland; 5 Department of Pediatrics, Sørlandet Hospital, Kristiansand, Norway; 6 Department of Occupational Medicine and Epidemiology, National Institute of Occupational Health, Oslo, Norway; 7 Institute of Health and Society, University of Oslo, Oslo, Norway; 8 Medical Birth Registry of Norway, Norwegian Institute of Public Health, Bergen, Norway; 9 Department of Global Public Health and Primary Health Care, University of Bergen, Bergen, Norway

**Keywords:** Employment, Behaviour problems, Child care, Child health, Work participation

## Abstract

This prospective population-based study examined associations between children’s behaviour problems and maternal employment. Information on children’s behaviour problems at 3 years from 22,115 mothers employed before pregnancy and participating in the Norwegian Mother and Child Cohort Study were linked to national register data on employment and relevant social background factors, mothers’ self-reported susceptibility to anxiety/depression and mother-reports of day-care attendance and fathers’ income. Mothers reporting their child to have severe (>2 SD) internalizing or severe combined behaviour problems (5 %) had excess risk of leaving paid employment irrespective of other important characteristics generally associated with maternal employment (RR 1.24–1.31). The attributable risk percent ranged from 30.3 % (internalizing problems) to 32.4 % (combined problems). Externalizing behaviour problems were not uniquely associated with mothers leaving employment.

## Introduction

The impact of maternal employment on children’s socio-emotional behaviour has been extensively studied with the bulk of the evidence convincingly indicating that maternal employment usually has no detrimental effect on small children’s behaviour, at least from child age 12 months (Lucas-Thompson et al. 2010; McMunn et al. 2010). In contrast to the abundance of studies exploring the impact of maternal employment on children’s behaviour, hardly any attention has been paid to the possible impact of children’s behaviour problems on maternal employment. This is despite previous research indicating that the majority of mothers adjust their work participation to meet family goals (Holmes et al. 2012; Mainiero and Sullivan 2005), making employment decisions in response to an intricate web of interconnected relational issues (Mainiero and Sullivan 2005), including their children’s health and behaviour (DeRigne 2012; Gordon et al. 2007).

Achieving and maintaining employment is difficult for mothers when children have special care needs (Baker and Drapela 2010; DeRigne 2012). A number of studies have documented associations between children’s care needs and lowered maternal employment rates (DeRigne 2012), with more mothers working part-time (Gordon et al. 2007; Hedov et al. 2002) despite no weaker desire for work (Gordon et al. 2007) and with the more severe conditions most strongly related to lower employment rates (DeRigne 2012; Montes and Halterman 2008). Children’s care needs have also been shown to hamper mothers’ further education, curtail their plans for future employment and inhibit their entry into new jobs (Booth and Kelly 1999; Shearn and Todd 2000). In addition, children’s care needs may reduce mothers’ work efforts and their ability to retain jobs, making it difficult to improve the quality of their jobs, wages and access to other work-related opportunities such as advancement (Coley et al. 2011). Childrearing and caregiving thus often require adjustments in terms of both time and money that may influence mothers’ career and long-term economic security severely. This may in turn have a number of negative consequences for maternal health and overall well-being. Over and above its obvious financial aspects, work provides additional benefits such as appreciation and social inclusion, a sense of purpose and belonging, learning, professional advancement, engagement, personal growth, and autonomy. Multiple roles, such as those of mother and professional have generally been found to afford beneficial outcomes (Barnett and Hyde 2001). Employment has for example been shown to reduce a sense of isolation and peripherality (Shearn and Todd 2000) and to relieve compromised health, stress and depression for maternal caregivers (Shearn 1998), whereas underemployment has been found to be associated with reduced life satisfaction and excess risk of depression and distress (Holmes et al. 2012).

Most studies to date have examined employment-related consequences for mothers of children with specific paediatric diagnoses involving intellectual impairments with low prevalence such as Down syndrome and autism (Baker and Drapela 2010), or disabilities more generally, including a wide range of different diagnoses (DeRigne 2012). Few have explored the employment-related impact of more prevalent conditions such as children’s behaviour problems despite such problems commonly introducing a host of family challenges and logistical burdens likely to interfere with parental employment opportunities, in particular for the mothers who are usually the primary caregivers (Baker and Drapela 2010; DeRigne 2012).

Behaviour problems in young children are common and usually load on two major dimensions described as internalizing (i.e., withdrawal, fearfulness, sadness, somatic problems) and externalizing (i.e., hyperactivity, inattention, aggression, defiance) factors (Achenbach et al. 1991). Distinct externalizing and internalizing symptom clusters have been shown from an early age and tend to precede later psychopathological problems (Gilliom and Shaw 2004). Symptoms from both clusters (internalizing and externalizing) may also co-occur from an early age of onset and comorbidity is high throughout childhood and adolescence (Angold et al. 1999; Gilliom and Shaw 2004; Lavigne et al. 1996). Depending on the definition, the overall prevalence of “problematic preschool behaviour” has been estimated to range between 10 and 25 % (SonugaBarke et al. 1997), and the prevalence of moderate to severe behaviour problems to be 8–10 % (Lavigne et al. 1996). Mothers of children with behaviour problems thus constitute a substantial proportion of the workforce.

This study explored associations between maternal employment and internalizing, externalizing and co-occurring internalizing/externalizing behaviour problems in toddlers. Data were comprised of questionnaire responses from a Norwegian prospective population-based birth cohort linked to national registry data on employment and demographic variables commonly associated with maternal employment in general including access to day care facilities, educational attainment, parity, partners’ income and age. The analyses were based on altogether 22,115 mothers all employed prior to birth. Data on education, marital status and parity were obtained from national registers. Additionally, maternal data on fathers’ income, day care attendance and the mothers’ own liability to anxiety/depression were accounted for.

## Method

### Sample

The present study was based on the Norwegian Mother and Child Cohort Study (MoBa) conducted by the Norwegian Institute of Public Health (Magnus et al. 2006; Nilsen et al. 2009). The MoBa study has been following pregnant women from the 17th week of pregnancy to the children’s 3rd birthday, with recruitment starting in 1999 and ending in 2008. The MoBa has been inviting pregnant women in Norway since 1999 and the goal of recruiting 100,000 pregnancies was reached in 2008. Recruitment occurred through a postal invitation when expecting mothers were scheduled for a routine ultrasound examination at 17–18 weeks of gestation (www.fhi.no/tema/morogbarn). The MoBa study was approved by the Regional Committee for Medical Research Ethics in south-eastern Norway and informed consent was obtained from each participant. At the first assessment (17–18 weeks of gestation), the participation rate was 38.5 % of those initially invited. The MoBa releases updated data files every summer. The current study is based on Version 6 of the quality-assured data files released for research in 2011.

The MoBa cohort is linked to the Medical Birth Registry of Norway, which contains standardized data regarding all pregnancies in Norway from 12 weeks of gestation (Irgens 2000). The registry contains a national identification number for all participants, allowing linkage with employment, benefit and income registries in the National Insurance Administration, the National Education Database of Statistics Norway, and the Central Population Register. This linkage provided longitudinal data for the children and their mothers, with updates for variables from these registries available through 2008.

### Exclusion and Inclusion Criteria

For mothers participating with more than one pregnancy in the study, only their first pregnancy ending in a live birth was included (*n* = 90,675). Participants with children born between 2006 and 2009 (*n* = 40,918) did not have sufficient information for their 3 year follow-up by 2008 and were thus not eligible for the study. Likewise, twins/triplets (1.9 %), participants who died or emigrated during the follow-up period (2.0 %), participants not previously employed (17.3 %) and participants with no partner at the time of recruitment (3.7 %) were not included, leaving altogether 37,503 eligible participants. Of these eligible participants, 40.9 % (*N* = 15,388) had not completed the questionnaire (Q6) used in the present analyses, thus leaving a total of 22,115 individuals.

### Measures

The main study exposures (i.e., internalizing and externalizing behaviour problems) in the child at age 3 were measured by maternal ratings on a set of items selected for the MoBa study to measure internalizing and externalizing behaviour problems in children. This set of items was selected by an expert group of researchers to represent a range of emotional and behavioural problems in 3 year old children. Items were culled from the widely used Child Behaviour Checklist (CBCL/1.5-5) (Achenbach 1992) and the Infant Toddler Social and Emotional Assessment (ITSEA) (Carter et al. 2003). The entire set of CBCL and ITSEA items selected for the MoBa to measure internalizing and externalizing problems in children were combined to obtain psychometrically stronger scales. Internalizing problems were measured by altogether 16 items (9 CBCL and 7 ITSEA items). The CBCL items reflected a broader grouping of three different domains of internalizing behaviour—anxiety, emotional reactivity, somatic problems, whereas the ITSEA items reflected general anxiety and separation distress. Cronbach’s alpha was 0.65 in the present sample. Externalizing problems were measured by 17 items in total (12 CBCL and 5 ITSEA items). The CBCL items reflected attention problems and aggression, whilst the ITSEA items reflected peer aggression and aggression/defiance. Cronbach’s alpha was estimated to be 0.83.

A two factor confirmatory factor analysis using Mplus fit the data moderately well (RMSEA = 0.048) and significantly better than a model specifying a single common factor ($\Updelta \chi^{2}_{1} = 7627.63,$ ΔAIC = −9717.723, ΔBIC = −9709.753). The correlation between the two factors was estimated to be 0.49.

Mothers rated the internalizing and externalizing items on a three point Likert scale with the categories “not true,” “somewhat or sometimes true” and “very true or often true.” We standardized mean sum scores on both scales for gender and transformed the scores into ordinal categories reflecting typical behaviour (reference), moderate behaviour problems (>1 SD above mean) and severe behaviour problems (>2 SD above mean), thus consistent with other research using the CBCL (Achenbach 1991; Eisenberg et al. 2001, 2009). As externalizing and internalizing behaviour problems tend to co-occur (Eisenberg et al. 2009) and the correlation between the two sum scales was 0.35, we constructed pure measures of internalizing and externalizing behaviour problems by excluding cases with co-occurring problems. Additionally, we constructed an index for co-occurring or combined internalizing/externalizing problems with three categories (1 = “neither,” 2 = “moderate internalizing/externalizing,” 3 = “severe internalizing/externalizing.”) To fulfil criteria for severe co-occurring problems, only one problem dimension had to be rated as severe.

Study outcome was employment status at child age 3 years among MoBa participants who were registered as part-time (i.e., 4–29 working hours weekly) or full-time (i.e., 30 h or more weekly) employed during the calendar year prior to childbirth. Employment status is reported annually by employers and recorded in the national employment register. A categorical variable indicating either (1) employed or (2) not employed (i.e., none or less than 4 h weekly) was constructed and used in the analyses.

Data on maternal age, educational attainment, parity and marital status at follow-up were provided by the NMBR, the Norwegian National Education Database of Statistics Norway and the Central Population Register. Education was coded into three categories (1 = “lower secondary school,” 2 = “upper secondary school,” 3 = “college/university”). Parity was similarly categorised into three groups (1 = “1 child,” 2 = “2 children” and 3 = “3 or more children”). Father’s income was collected from mother report at child age 3 years. Annual income above 500,000 NOK (about 90–100,000 US dollars) was coded as high, income below 200,000 as low and intermediate income as average. Day care attendance was similarly mother-reported at child age 3 years.

The mothers’ susceptibility to anxiety/depression was measured by a 5-items version of the 25-item Hopkins Symptom Checklist SCL-25 (Hesbacher 1980). The SCL-5 used here has been shown to perform similarly to the longer version (Tambs and Moum 1993), and is suitable for detecting psychological problems, specifically symptoms of anxiety and depression in non-psychiatric settings. The SCL was initially designed as a “state” measure, but a range of studies have demonstrated that common psychological symptoms display considerable temporal stability, to a large extent reflect stable or “trait”-like aspects, largely due to genes (Nes et al. 2007). Susceptibility to anxiety/depression has also been shown to influence maternal ratings of children’s behaviour problems (De Los Reyes and Kazdin 2005; Müller et al. 2011; Richters 1992). More specifically, maternal levels of self-reported depression have been shown to influence maternal ratings of children’s emotional and behavioural problems consistent with the depression-distortion hypothesis (Richters 1992). This hypothesis suggests that depression promotes a negative bias in how mothers perceive their children’s behavior and emotional problems. We therefore used the mothers’ SCL-scores from gestation week 17 as a proxy for their stable vulnerability to anxiety/depression. Mothers completing the SCL-5 were asked to indicate on a 1-point scale if they during the last 14 days had been bothered at all, was a little bit, quite much, or very much bothered by problems such as “Feeling blue” and “Worrying too much about things.” Cronbach’s alpha was 0.77. Mean sum scores were computed and a score of 2.0 was used as a clinical cut-off point according to convention (Strand et al. 2003). This cut-off point has been shown to constitute a valid indicator of mental disorder with prediction being somewhat better for depression than other mental disorders (Strand et al. 2003).

### Statistical Analysis

Analyses were conducted using Stata/SE version 12 (2011). As non-employment among mothers of small children is fairly common, logistic regression analyses and corresponding odds ratios (OR) may be misleading, overestimating the prevalence ratio (Barros 2003; Mcnutt et al. 2003). We therefore performed robust Poisson regression analyses which provide more interpretable prevalence ratios and have the additional advantage of providing estimates that are relatively robust to omitted covariates (Neuhaus and Jewell 1993; Zou 2004). Crude effects of internalizing and externalizing behavior problems on maternal employment at child age 3 years adjusted for birth year (2000–2005) were estimated and then further adjusted for potential confounding influences by sequentially entering five blocks of variables (susceptibility to anxiety/depression, age, educational attainment, father’s income, and family size, the latter block (family size) based on parity and marital status at follow-up). These factors have all been shown to influence maternal employment. The regression analyses were performed on gender-specific z-scores and the results presented as adjusted relative risk (RR) ratios with corresponding 95 % confidence intervals (CI) for being out of employment at child age 3 years. To estimate how much of maternal non-employment that could be attributed to their child’s behaviour problems, the attributable risk and the attributable risk percent with corresponding 95 % confidence intervals were calculated.

## Results

### Attrition

Comparisons of the employment status of the 59 % mothers who returned the questionnaire at child age 3 years (responders) with the remaining 41 % mothers who did not return the questionnaire (non-responders) indicated that the non-responders had negligibly higher risk of non-employment at follow-up (83.3 % employed) than responders (84.7 % employed) and although minor, the difference was significant (χ² = 12.74, df = 1, *p* < 0.0001).

### Descriptives

Table 1 shows employment status at follow-up for the different groups of mothers. A total of 15.3 % (*n* = 3,385) of the sample were not employed at follow-up, 38.6 % (*n* = 8,540) were part-time employed and 46.1 % (*n* = 10,190) full-time employed. Altogether 25.5 % of the mothers reported their child to have moderately to severely elevated levels of behaviour problems. More specifically, 16.2 % of the mothers reported their child to have either moderate internalizing or moderate externalizing problems, 2.2 % reported moderate combined internalizing/externalizing problems, 4.3 % reported either severe internalizing or severe externalizing problems, and 2.7 % reported their child to have combined problems of which at least one domain of behaviour problems (i.e., internalizing or externalizing) was rated as severe. Thus, in total 7 % of the mothers reported their child to have severe behaviour problems (i.e., pure internalizing, pure externalizing or combined internalizing/externalizing). Internalizing and externalizing problems co-occurred considerably, with approximately 30 % of cases (i.e., those scoring above 1 SD) presenting combined problems.

**Table 1:** Maternal employment status according to type and severity of behaviour problems

Child behaviour problems	Number	Maternal employment		
		No (%)	Part-time (%)	Full-time (%)
Internalizing				
No	16,484	14.8	38.8	46.4
Moderate	1,627	16.3	37.4	46.3
Severe	500	21.2	39.2	39.6
Externalizing				
No	16,484	14.8	38.8	46.4
Moderate	1,965	15.0	38.6	46.4
Severe	456	16.5	36.4	47.2
Combined internalizing/externalizing				
No	16,484	14.8	38.8	46.6
Moderate	484	15.9	36.6	47.5
Severe	599	21.9	39.1	39.1

For internalizing problems and combined internalizing/externalizing problems, the percentage of mothers who had left paid employment at follow-up increased with problem severity (Table 1). There were no significant differences in part-time employment across groups of mothers. Most children attended day care (95.4 %) and day care attendance was neither associated with children’s internalizing behaviour problems (χ² = 4.12, df = 2, *p* = 0.127), externalizing behaviour problems (χ² = 1.55, df = 2, *p* < 0.46), or combined behaviour problems (χ² = 2.97, df = 2, *p* = 0.226). Table 2 provides information on socio-demogratic characteristics according to employment status. Young age (<24 years), low educational attainment (lower secondary school), low income (lowest quartile) and mental health problems during pregnancy were all significantly associated with leaving paid employment by child age 3 years. Likewise, mothers who were single at follow-up (i.e., had separated/divorced since recruitment) were more likely to have left the workforce by child age 3 years, than mothers having a marital or equivalent partner.

**Table 2:** Socio-demographic characteristics of mothers and the children according to employment status

Category	Number	Not employed (%)
Child sex		
Female	10,887	15.2
Male	11,228	15.4
Mother’s age		
24 years and younger	1,676	27.5
25–29 years	7,829	16.3
30–34 years	8,974	13.3
35 years and older	3,636	12.7
Maternal educational attainment		
Lower secondary education	1,891	26.1
Upper secondary education	5,855	19.5
College/university	14,369	12.5
Father’s income at follow-up		
Low	1,939	21.6
Average	16,348	14.3
High	2,691	16.1
Household type at follow-up		
Two-parent	21,103	15.0
One-parent	1,012	21.4
Number of children at follow-up		
1	4,559	12.3
2	12,612	15.8
3 or more	4,944	16.8
Maternal liability to anxiety/depression in pregnancy		
No	21,022	14.8
Poor	1,093	24.3

### Regression Analyses

Results from the regression analyses are tabulated in Table 3 (internalizing behaviour problems), Table 4 (externalizing behaviour problems) and Table 5 (combined behaviour problems). The regression analyses showed that the impact of having a child with severe internalizing behaviour problems on maternal non-employment was moderate (RR 1.45, 95 % CI 1.22–1.72), and significant also after adjusting for a wide range of important covariates known to affect employment in general (RR 1.31, 95 % CI 1.10–1.55), including susceptibility to common mental disorders (Table 3). By contrast, the crude effect of severe externalizing behaviour problems, was not significantly associated with maternal non-employment (RR 1.11, 95 % CI 0.90–1.37). Combined internalizing/externalizing problems were significantly related to non-employment at follow-up if severe (RR 1.48, 95 % CI 1.26–1.72) and still significant in the fully adjusted model (RR 1.23, 95 % CI 1.06–1.44). Combined behaviour problems of moderate severity (between 1 and 2 SD above mean) were not significantly associated with mothers leaving the work force (RR 0.99, CI 0.81–1.23).

**Table 3:** Crude and adjusted associations of child internalizing behaviour problems with maternal employment at child age 3 years

	Pure internalizing behaviour problems			
	Moderate		Severe	
	RR	95 % CI	RR	95 % CI
Crude effect of internalizing problems	1.11	0.98–1.24	1.45	1.22–1.72
Stepwise inclusion of covariates				
+ Maternal liability to common mental disorder	1.09	0.97–1.22	1.39	1.17–1.65
+ Maternal age	1.07	0.95–1.20	1.34	1.13–1.60
+ Educational attainment	1.06	0.95–1.19	1.29	1.09–1.53
+ Father’s income	1.06	0.95–1.19	1.29	1.08–1.53
+ Marital status and number of children	1.09	0.97–1.22	1.31	1.10–1.55

*RR* risk ratio, *CI* confidence interval, Birth year (2000–2005) is adjusted for in all analyses

**Table 4:** Crude and adjusted associations of child externalizing behaviour problems with maternal employment at child age 3 years

	Pure externalizing behaviour problems			
	Moderate		Severe	
	RR	95 % CI	RR	95 % CI
Crude effect of externalizing problems	1.01	0.91–1.13	1.11	0.90–1.37
Stepwise inclusion of covariates				
+ Maternal liability to common mental health	1.00	0.90–1.12	1.09	0.88–1.34
+ Maternal age	0.98	0.88–1.10	1.05	0.85–1.29
+ Educational attainment	0.96	0.86–1.07	1.02	0.83–1.25
+ Father’s income	0.97	0.87–1.08	1.02	0.83–1.26
+ Marital status and number of children	0.97	0.87–1.08	1.03	0.84–1.27

*RR* risk ratio, *CI* confidence interval, Birth year (2000–2005) is adjusted for in all analyses

**Table 5:** Crude and adjusted associations of child combined internalizing/externalizing behaviour problems with maternal employment at child age 3 years

	Combined behaviour problems			
	Moderate		Severe	
	RR	95 % CI	RR	95 % CI
Crude effect of externalizing problems	1.09	0.89–1.34	1.48	1.26–1.72
Stepwise inclusion of covariates				
+ Maternal liability to common mental health	1.07	0.87–1.32	1.39	1.19–1.63
+ Maternal age	1.01	0.82–1.24	1.32	1.13–1.54
+ Educational attainment	0.97	0.79–1.20	1.23	1.05–1.42
+ Father’s income	0.98	0.81–1.23	1.22	1.05–1.42
+ Marital status and number of children	0.99	0.81–1.23	1.23	1.06–1.44

*RR* risk ratio, *CI* confidence interval, Birth year (2000–2005) is adjusted for in all analyses

Low maternal age, low educational attainment, low income, and vulnerability to poor mental health were all potent risk factors for non-employment (data not shown).

### Attributable Risk

The attributable risks among mothers of children with severe internalizing and severe combined behavior problems were 6.4 and 7.9 per 100 mothers, respectively. The attributable risk per cent was 30.29 (95 % CI 15.3–45.3) for severe internalizing problems and 32.43 (95 % CI 19.1–45.8) for severe combined problems, indicating that among mothers of children with severe internalizing or severe combined behavior problems, nearly one third of their employment drop-out was attributable to the child’s behavior problems.

## Discussion

This study indicates that internalizing behaviour problems and co-occurring internalizing/externalizing behaviour problems in toddlers constitute independent risk factors for previously employed mothers to leave paid employment by child age 3 years—at least temporarily. A total of 5 % of mothers report their children to display severe internalizing or severe combined behavior problems. Of these mothers, 21–22 % are not in paid employment at follow-up as compared to 14.8 % of those having children with no behaviour problems. Children’s behaviour problems thus appear to play an important role in their mother’s employment decisions and career development.

These results are in line with findings from a previous study showing difficult temperament (i.e., negative mood, high intensity of reactions, low adaptability, withdrawal responses, low rhythmicity) in infancy and toddlerhood to be related to lower maternal employment during the toddler and preschool age (Galambos and Lerner 1987). Behaviour problems in adolescents have also been shown to predict poor mothers’ labour market success (Coley et al. 2011). Specifically, behaviour problems were shown to constitute one piece of a cumulative package of challenges that disadvantaged women face when accessing and retaining stable and good quality employment. The present study explores the association of maternal employment with child behaviour problems in toddlers only. However, there is incontrovertible evidence of continuity between toddler’s behavioral and socio-emotional problems and later psychopathology (Briggs-Gowan and Carter 2008; Caspi et al. 1996; Keenan et al. 1998). The current and previous findings thus suggest that children’s behavior problems may have a sustained impact on maternal employment.

The findings are remarkable in view of the fact that Norwegian children have access to high quality day care from age 1 year. Commonly, care responsibilities are reported to influence parents’ employment decisions when their children have emotional and behavioural disorders (Rosenzweig et al. 2008). For example, non-Scandinavian studies (e.g., American) indicate that access to child care and supports specific to the needs of their children, are essential for parents of children with emotional or behavioural disorders to reduce stress and promote work-life integration (Rosenzweig et al. 2008). In Scandinavian countries, quality child care mostly corresponds to the existing demand and the large majority of children in this study (>95 %) are attending kindergarten. In addition, maternal employment status is not associated with day care attendance. Thus, quality day care facilities do not eliminate the risk of maternal drop-out from the workforce.

Most likely, lower employment among mothers of children with behaviour problems is attributable to multiple factors. Parental time is commonly regarded as a key determinant in healthy child development and there is still a gendered dimension with respect to the amount and quality of time that mothers and fathers spend with their children (Monna and Gauthier 2008). A general change in social norms and expectations concerning parenting and children’s needs has been suggested, with the emergence of a culture characterized by more intensive mothering (Moro-Egido 2012). Both employed and non-employed mothers are for example reported to have increased the time they devote to their children and mainly through active, quality time with time-intensive children below 6 years (Moro-Egido 2012). Increased focus on quality parenting, juggling of regular family duties and work demands, along with the additional challenges introduced by a child’s behaviour problems are likely to overload the mother. Children suffering from behavioural, emotional and psychosocial problems demand enhanced parental attention and supervision (Bernheimer et al. 2003; Coley et al. 2011), they may divert attention from other important aspects of family functioning (e.g., other children, parental relationship) and are likely to bring a host of logistical burdens such as time-consuming visits to medical and social services. Of note, these factors are associated with both externalizing and internalizing behaviour problems. In this study, however, internalizing and co-occurring behaviour problems, but not externalizing behaviour problems, are associated with maternal non-employment at child age 3 years. A possible explanation for this discrepancy may be related to differential maternal responses to small children’s sadness, wariness and somatic symptoms on the one hand side, and aggression and defiance on the other. Parents commonly perceive externalizing behaviour problems as annoying, noxious and stressful and children with externalizing behaviour problems tend to receive more discipline and control from their parents (Moffitt 2005). Interestingly, adoption studies indicate that the link from children’s genetic risk of externalizing behaviour problems to adoptive parents parenting is mediated by the child’s aggressive behaviour problems (Moffitt 2005; O’Connor et al. 1998). Thus, children’s externalizing behaviour problems elicit more punitive parenting. By contrast, internalizing behaviour problems are typically regarded as more subtle and less aversive, are less likely to evoke punitive responses and more likely to be associated with over-involved, over-solicitous and over-protective maternal responses (Degnan et al. 2010). Genetically informative research indicates that these maternal responses primarily are elicited by the child’s genotype (Narusyte et al. 2008). That is, mothers experiencing their children as depressed, anxious or withdrawn become more emotionally overinvolved in their parenting (i.e., evocative gene environment correlation). The resulting amalgam of stressors contributing to elevate parenting pressures and subjective stress may be experienced as incompatible with occupational requirements and loyalty, causing mothers to leave paid work—at least temporarily. Mothers of children with internalizing or combined behaviour problems are therefore likely to become increasingly disadvantaged with regard to future employment prospects, income opportunities and pension benefits. Along with erosion of qualifications, extended career breaks may also lead to reduced self-esteem and a feeling of isolation, making return to work increasingly difficult. These employment-related consequences may exacerbate the health-related consequences of maternal caregiving (Brehaut et al. 2009; Hill et al. 2010; Singer 2006). The work place constitutes one of the primary social worlds of the twenty-first century adult and has been shown to provide a number of health-related benefits to individuals (Ahrens and Ryff 2006; Headey et al. 2010), mothers in general (Egger and Angold 2006; Rosenzweig et al. 2008), mothers of children with special care needs (Shearn and Todd 2000; Shearn 1998) and mothers of children with behavioural problems more specifically (Morris 2012). Even mothers who do not desire employment may accrue mental health benefits from paid employment, at least if they acquire high quality jobs (Usdansky et al. 2012). Leaving paid work may thus have far-reaching and negative long-term consequences for these mothers in terms of both financial and labour market matters as well in terms of health and well-being—of relevance to themselves (Ahrens and Ryff 2006; Bailey et al. 2007; Holmes et al. 2012; Munk-Olsen et al. 2006), their child (McMunn et al. 2010) and the work place (Hill and Weiner 2003; Mainiero and Sullivan 2005).

### Strengths and Limitations

To our knowledge, this is the first study to investigate associations between toddlers’ behaviour problems and maternal employment. The main strengths of the study include its large sample drawn from a population-based study and its prospective design linking cohort data to registry-based information on employment and demographic data. Although previous studies have found associations between child disabilities and maternal employment, most have used cross-sectional designs, parental reports only, or have not included an appropriate reference group (DeRigne 2012; Gordon et al. 2007). An adequate reference group of mothers of healthy children of a comparable age is vital to explore the independent effects of child condition on maternal employment. Mothers of small children tend to be less stable in their job-holding and working hours than women with older or no children and different developmental stages are likely to introduce different caretaking challenges, particularly for children with special needs.

However, the results also need to be interpreted in the context of some additional limitations. First, despite being able to contrast the risk of non-employment for mothers of children with behaviour problems with the risk for mothers of typical children of comparable age, we cannot describe the processes that mediate between the child’s behaviour problems and the mother’s decision to leave employment.

Secondly, the participation rate in the MoBa study is lower than optimal and mothers of higher age, living in stable relationships and having healthy outcomes are over represented. The overall employment rate (84.7 %) corresponds fairly well with that of women with young children in the general population (Bø et al. 2008) and participating mothers at child age 3 years (59 %) did not differ substantially from those who ceased to participate (41 %) with respect to employment status at follow-up. Still, the pres-ence of a selection bias in terms of better educated respondents, probably causes an underestimation of the prevalence of exposure and renders the association observed between children’s behaviour problems and maternal non-employment conservative.

Third, the analyses were based on a selection of items measuring externalizing and internalizing problems from two separate assessment instruments (i.e., CBCL and ITSEA) and Cronbach’s alphas were moderate. The two assessment instruments are strongly overlapping with respect to the underlying concepts (i.e., internalizing and externalizing). Nevertheless, use of shorter, less reliable scales has reduced rather than increased our ability to detect associations between child behaviour problems and maternal employment.

Fourth, we adjusted for the mothers’ vulnerability to common mental disorders reported during pregnancy. Maternal psychopathology may be related to childhood internalizing and externalizing problem behaviour by multiple factors such as shared genes, social learning, maternal insensitivity and inadequate parenting. Higher maternal ratings may therefore reflect a truly higher level of problem behaviour in these children. However, when running the analyses in subsets of mothers with and without risk of mental health problems, associations between exposure and outcome were practically identical.

Scandinavian countries differ from most other countries in that women’s total work participation is high, part-time employment is of comparable social status to full-time employment, and quality child care is generally available (Haas 1996 and Ugreninov 2012). Additionally, governmental sponsored health care is available to all citizens. The results may therefore not extrapolate to all countries.

Lastly, the information level is moderate and inferences must therefore be made with caution.

## Conclusions

The present study shows that mothers’ of young children with internalizing or co-occurring internalizing/externalizing behaviour problems to a greater degree leave paid employment than mothers in general. Children’s behavior problems thus entail a range of immediate and long-term consequences for the financial and probably overall well-being of their mothers. This comes in addition to the loss of human capital and talent for companies and organizations. Previous research indicates that employees with access to flexible workplace options show greater commitment to their organization, and the greatest when flexibility in both time and place are combined, with the most obvious benefits for women with children below five years (Hill et al. 2010). Flexibility in time and place may also be vital for mothers’ decisions and opportunities to remain employed. Further studies are needed to gain a better understanding of the interrelationship between maternal employment and children’s socio-emotional behaviour to promote targeted policies to enable mothers, families and organizations to thrive.
